# Right paraduodenal hernia, classification, and selection of surgical methods: a case report and review of the literature

**DOI:** 10.1186/s13256-023-04286-1

**Published:** 2023-12-30

**Authors:** Xiao-Long Wang, Gui-Xiu Jin, Jian-Feng Xu, Zhi-Rong Chen, Li-Meng Wu, Zhi-Long Jiang

**Affiliations:** 1 Department of General Surgery, Taixing People’s Hospital, No. 1, Changzheng Road, Taixing, Jiangsu China; 2 Department of Gynecology and Obstetrics, Taixing People’s Hospital, Taixing, Jiangsu China; 3 Department of Radiology, Taixing People’s Hospital, Taixing, Jiangsu China; 4 Department of General Surgery, The First Affiliated Hospital of Soochow University, Suzhou, Jiangsu China

**Keywords:** Right paraduodenal hernia, Internal hernia, Surgical methods, Mesocolic hernia

## Abstract

**Background:**

Considering that right paraduodenal hernia is a rare internal hernia with abnormal anatomy and is often encountered during an emergency, surgeons may lack knowledge about it and choose incorrect treatment. Thus, this case report is a helpful complement to the few previously reported cases of right paraduodenal hernia. Additionally, we reviewed all the reported right paraduodenal hernia cases and proposed appropriate surgical strategies according to different anatomical features.

**Case presentation:**

The case involved a 33-year-old Chinese male patient who was admitted to the hospital due to abdominal pain. The patient was initially diagnosed with small bowel obstruction, and conservative treatment failed. An emergency operation was arranged, during which a diagnosis of right paraduodenal hernia was made instead. After surgery, the patient recovered well without abdominal pain for 2 years.

**Conclusion:**

Although right paraduodenal hernia accounts only for a small proportion of paraduodenal hernia, its anatomical characteristics can vary considerably. We divided right paraduodenal hernia into three types, with each type requiring a different surgical strategy.

## Introduction

Paraduodenal hernias (PDHs) are congenital internal hernias. The incidence of PDH is unclear because some patients remain asymptomatic throughout their lives. According to autopsy results, the incidence of internal hernias is between 0.2% and 0.9%, while PDH accounts for approximately 53% of internal hernias [1–5]. The low incidence of PDH has also been demonstrated in autopsies [6–8]. PDH can be divided into left and right PDH according to completely different embryological and anatomical features [9, 10]. Right PDH is less common than left PDH, with a 1:3 ratio [1, 2, 11–15].

In clinical practice, a considerable number of patients with right PDH do not receive an accurate diagnosis preoperatively because right PDH is rare and most commonly presents as an emergency [5]. During surgery, appropriate surgical methods could not be adopted timely due to unfamiliarity with the anatomy of right PDH, resulting in prolonged operation and poor recovery of the patient. In 2021, we encountered a case of right PDH that was diagnosed intraoperatively. After the operation, we researched the literature and found that there was no article summarizing right PDH based on anatomic features. Additionally, in many reports, the Ladd’s procedure was used without choosing an appropriate surgical method on the basis of the anatomy of right PDH. Therefore, we aimed to classify right PDH according to its anatomical and etiological features. According to this classification, different types of surgery can be selected.

## Materials and methods

We found nearly 500 literature reports on PDH using the keywords “paraduodenal hernia,” “para-duodenal hernia,” “Treitz's hernia,” and “mesocolic hernia” in PubMed. After an initial analysis of the literature, only articles with right PDH confirmed by surgery and with imaging or anatomical illustrations from the literature explaining anatomical features were included in our study. We excluded cases in which right PDH was not surgically confirmed and anatomical features could not be identified as well as studies in languages other than English and published before 1970. Furthermore, three studies were excluded due to missing full text. Along with the cases reported in the literature, right PDH encountered in our hospital was also included and presented as a case report.

All cases included an obtained informed consent signed by patients. The research protocol complied with the ethical standards of the Institutional Research Committee and the 1964 Declaration of Helsinki and its subsequent amendments. As this is a retrospective observational study and review of the literature, no formal consent to this study was required, nor approval by the Institutional Research Committee.

## Case report

**Fig. 1:**
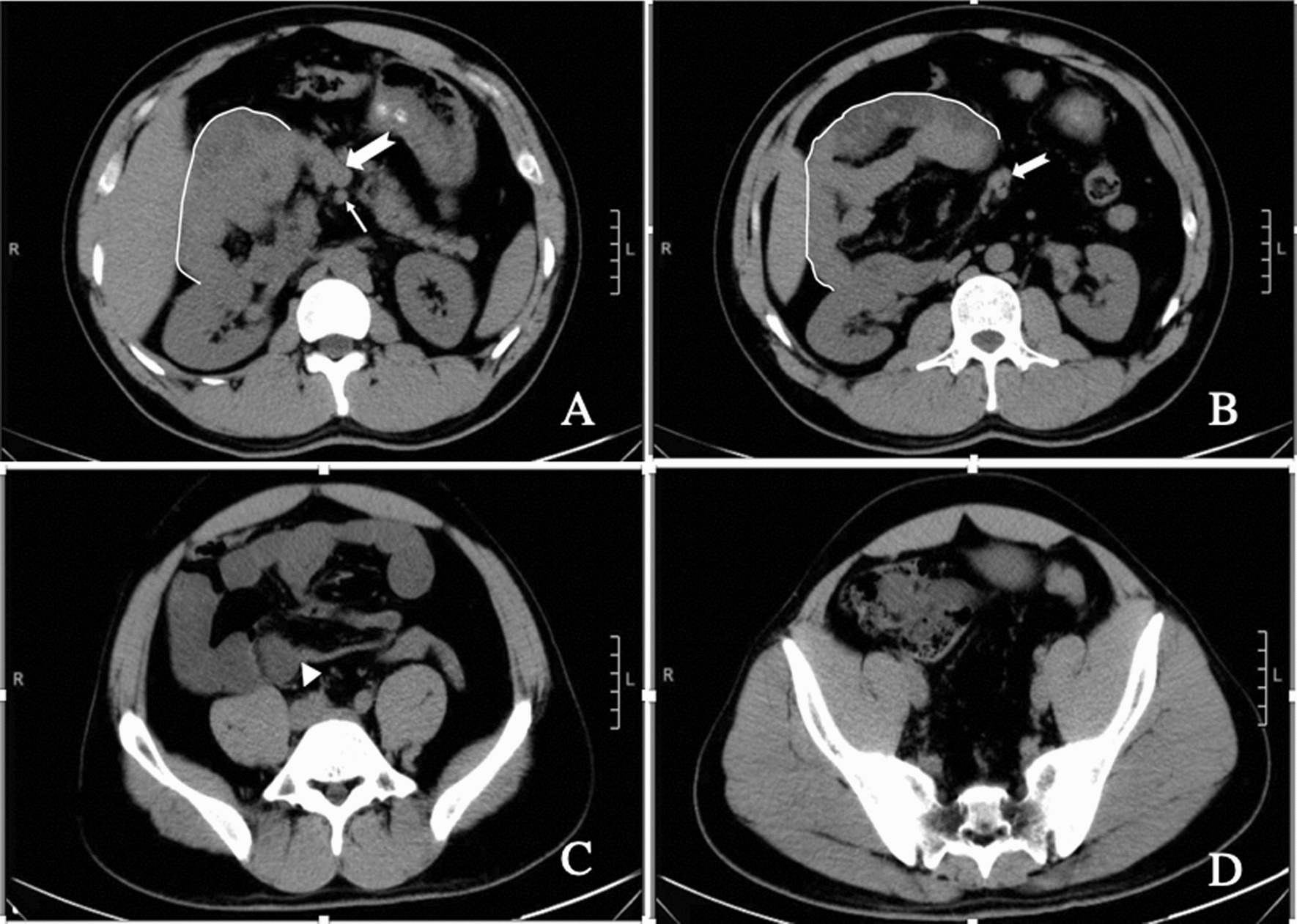
White line: hernia sac. Long arrow: superior mesenteric artery. Dovetail arrow: superior mesenteric vein and its branches. Triangle: the obstruction site. **A** Superior mesenteric vein (SMV) and superior mesenteric artery (SMA) arranged anteroposteriorly; there was no horizontal part of the duodenum on the dorsal side of the superior mesenteric vessels. **B** Jejunal vessels originate from the left side of the SMV and then turn to the right side of the abdomen. **C** White arrow indicates the obstruction site. **D** There was a paucity of small bowel loops in the pelvis. The cecum was located in the right lower quadrant

A 33-year-old Chinese male patient presented to our emergency department with 4 hours of acute abdominal pain. The patient reported no nausea, vomiting, or other associated symptoms. He had no medical history of abdominal discomfort or abdominal surgery. Physical examination revealed tenderness in the right lower quadrant with rebound tenderness and active bowel sounds. In the emergency department, computed tomography (CT) was performed. The interim report indicated that a clumpy, “C”-shaped abnormal intestinal shadow was observed in the right mid-upper abdomen, with increased and entangled mesenteric density in the surrounding adipose space, suggesting the possibility of internal abdominal hernia (Fig. 1).

**Fig. 2:**
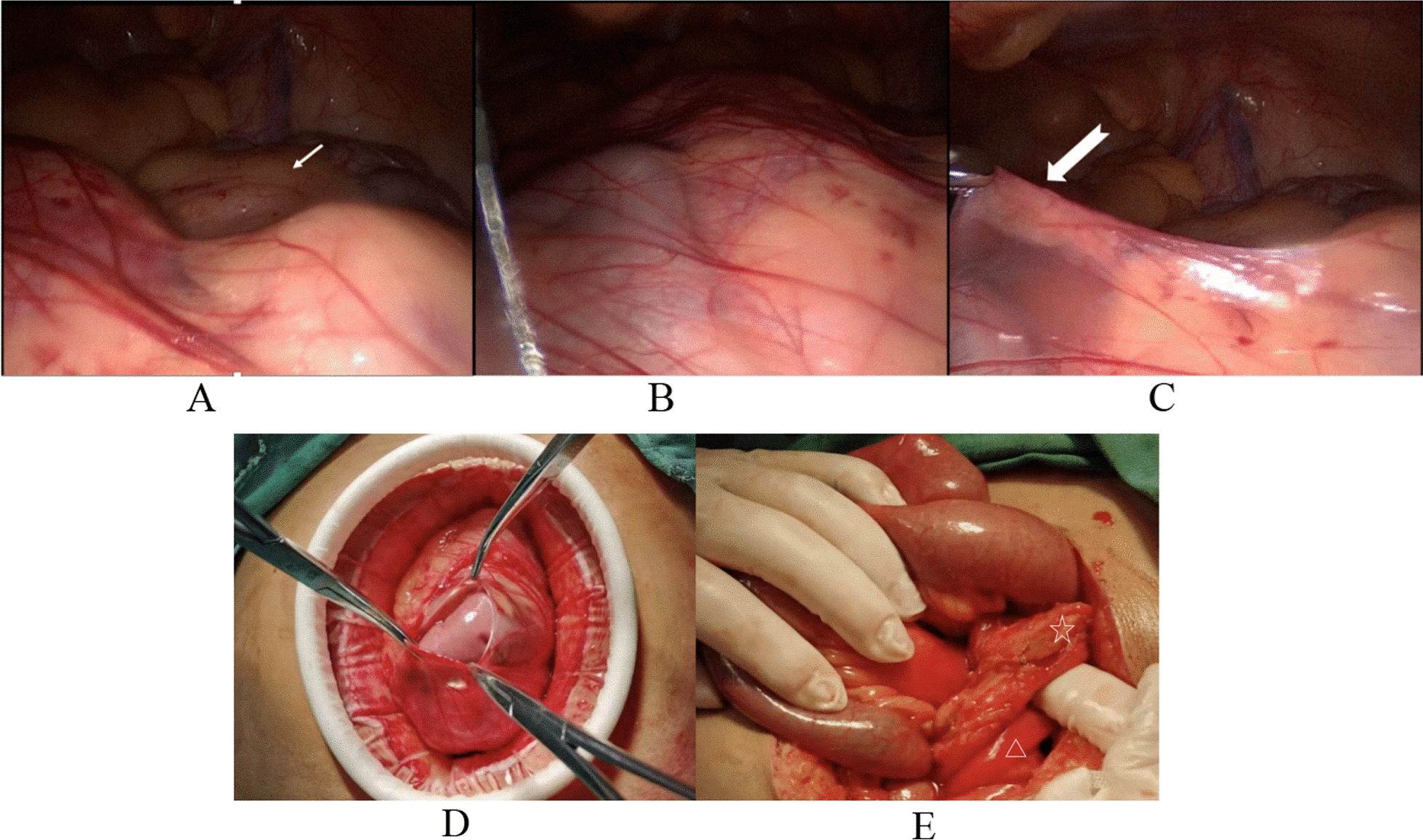
Long arrow: the cecum. Dovetail arrow: the hernia sac. Triangle: the efferent loop. Star: superior mesenteric vessels and surrounding tissues. **A** The cecum in its normal position. **B** and **C** The original appearance of the hernia sac and the appearance after it was lifted. **D** and **E** The hernia sac (ascending mesocolon) was opened by mistake

**Fig. 3:**
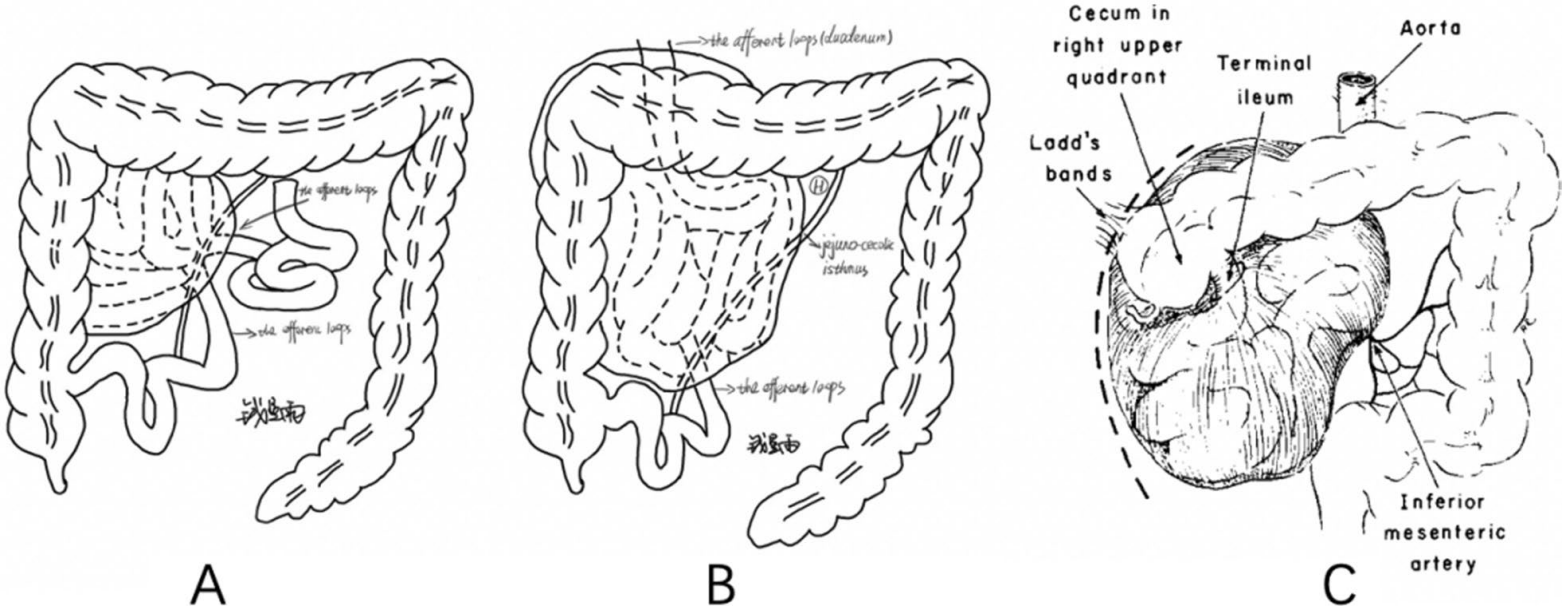
**A** The first type of right paraduodenal hernia. **B** The second type of right paraduodenal hernia. **C** The third type of right paraduodenal hernia [45] (reprinted by permission from Copyright Clearance Center provided by Elsevier: The American Journal of Surgery, 1974. 128(3): p. 358–361)

**Fig. 4:**
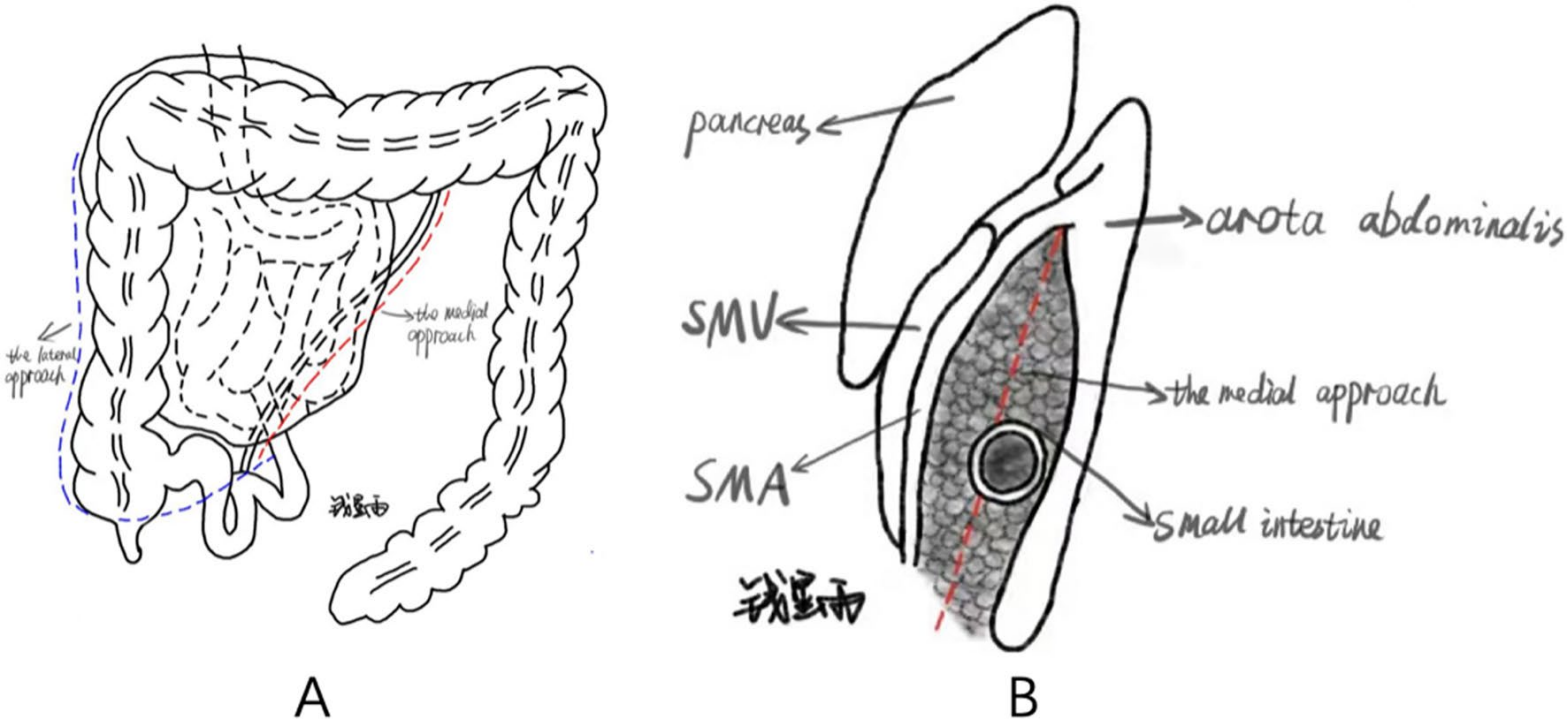
**A** The blue dashed line indicates the lateral approach; the red dashed line indicates the medial approach. **B** Left view of the medial approach

After admission, the patient received conservative treatment for 4 hours, but no relief of abdominal pain was observed. After consulting his family members, an emergency operation was agreed upon. Laparoscopy was first used to assess the entire abdomen. We found that the third segment of the duodenum, jejunum, and most of the ileum were located in a clear capsule (Fig. 2). The cecum was located in the right lower quadrant, and 25 cm from the ileocecum, a segment of the small bowel of about 5 cm was compressed beneath the superior mesenteric vessels. We then decided to switch to open surgery. We cut the unvascularized area (the jejuno-cecolic isthmus) (Fig. 3B) on the dorsal side of the superior mesenteric vessels (Fig. 4B), cephalic to the root of the superior mesenteric vessels. The orifice of the hernia sac was fully opened. During the operation, the anterior wall of the hernia sac (ascending mesocolon) was opened by mistake and then completely repaired with absorbable sutures. The patient’s postoperative course was uneventful. After 2 years of follow-up, the patient reported no abdominal discomfort.

## Results of literature review

We identified a total of 500 records from the database. First, we performed a rough screening by reading abstracts to exclude patients with left PDH. Then, we read the full text carefully, excluding cases with a history of previous abdominal surgery, other abdominal diseases, and unclear descriptions, finally including 34 cases. The ratio of male to female patients was about 21:12. The median age was about 29 years. The number of patients with disease presenting after the age of 50 years was extremely small. The emergency surgery rate was approximately 60%. Afterward, we categorized all cases. The classification is based on anatomical characteristics combined with the theory of embryonic development, as detailed in the Discussion section. Table 1 demonstrates that surgical options for each type of right PDH are different. Classifying cases can allow clinicians to become familiar with this disease and choose a reasonable surgical method.

**Table 1:** Published cases of surgically confirmed right PDHs

No.	Author	Time	Patient	Emergency^a^	Surgery	Type	SO^b^
1	Tadaomi Fukada *et al*. [16]	2010	46/M	Y	Close the orifice	2(H)	Y
2	Marisa E. Schwab *et al*. [17]	2022	84/M	Y	Ladd’s procedure	3	
3	Mohammed Hassan *et al*. [18]	2012	19/M	Y	Close the orifice	1	
4	Bao-Lin Liu *et al*. [19]	2010	43/M	Y	The medial approach^c^	1	
5	N. Peru *et al*. [20]	2020	29/M	Y	Ladd’s procedure(s)^d^	2	
6	Michelle Ong [21]	2017	53/F	Y	Widen hernia orifice	2	
7	L.J. Yeoman *et al*. [22]	1994	27/M	Y	Unclear	1	
8	David M. Warshauer *et al*. [23]	1992	32/M	Y	Unclear	2	
9	Enrico Erdas *et al*. [24]	2014	32/F	Y	Close the orifice	2(H)	
10	Venkatraman Indiran *et al*. [25]	2016	19/M	Y	Ladd’s procedure(s)	2	
11	Rahul, K. *et al*. [26]	2013	11/M	N	Ladd’s procedure	3	
12	E. Antedomenico *et al*. [27]	2004	24/F	N	Ladd’s procedure(s)	2	
13	John Mathew Manipadam *et al*. [28]	2018	31/F	N	Ladd’s procedure(s)	1	
14	James G. Bittner IV [6]	2009	26/F	unclear	Ladd’s procedure(s)	1	
15	Stephanie Walkner *et al*. [29]	2019	37/M	N	Ladd’s procedure(s)	2	
16	S. Rajesh *et al*. [30]	2015	Unclear	Unclear	Ladd’s procedure	3	
17	Ramnik V. Patel *et al*. [31]	2013	1W/M	Y	Ladd’s procedure	3	
18	Kapil Rampal *et al*. [32]	2022	26/M	Y	Ladd’s procedure(s)	2	
19	H.Y. Yoo *et al*. [33]	2000	19/F	Unclear	Close the orifice	2(H)	
20	Thomas B. Coopwood *et al*. [34]	1972	42/F	Unclear	Close the orifice	1	
21	Chien-Heng Lin *et al*. [14]	2008	15/M	Unclear	Unclear	2	
22	Viktoria Lamprou *et al*. [35]	2022	37/F	Y	Close the orifice	2(H)	
23	Takahiro Tomino *et al*. [36]	2015	23/M	Y	Close the orifice	1	
24	Vijay Anand Ismavel *et al*. [37]	2021	23/M	Y	Unclear	1	
25	Andrew S. Olearchy *et al*. [38]	1979	27/M	Y	Widen the orifice	1	
26	Yen-Hsiu Liao *et al*. [39]	2011	45/F	Y	Close the orifice	1	
27	Navin Poudel *et al*. [40]	2021	36/M	N	Ladd’s procedure(s)	2	
28	Asif Abdullah *et al*. [41]	2010	48/F	Y	Ladd’s procedure(s)	2	
29	Basilios Papaziogas *et al*. [42]	2004	17/M	Y	Close the orifice	1	
30	T.K.Y. Lee *et al*. [43]	1990	29/M	N	Widen the orifice	2	
31	Tsuyoshi Shinohara *et al*. [12]	2004	12/M	Y	Ladd’s procedure(s)	1	
32	M. A. Meyers [15]	1970	50/F	Unclear	Unclear	1	
33	Takeyama, N. *et al*. [1]	2005	31/M	Y	Unclear	1	
34	Kwan, B. [44]	2020	18/F	Unclear	Close the orifice	2(H)	

^a^Emergency indicates emergency surgery

^b^SO indicates secondary surgery

^c^The medial approach indicates the approach described in Fig. 4

^d^Ladd’s procedure(s) is described as the separation of the ascending colon and the cecum along the white line of Toldt, with the medial side ensuring that the hernia sac opening is reached. It is not the standard Ladd’s procedure, but it was expressed as Ladd’s procedure in many reports. This paper uses the lateral approach, as shown in Fig. 4, which is a way to enlarge the orifice of the hernia sac. The lateral approach is used in place of the Ladd’s procedure(s) in the following section

## Discussion

At present, it is generally accepted that PDH is related to changes in peritoneal fixation and vascular folds. Most literature ascribes the pathogenesis of right PDH to the malrotation of the prearterial limb of the midgut loop [5, 6, 9, 10, 12, 24, 28, 45–47], which is an incomplete speculation. Since right PDH is a rare disease, and most reports are case reports only, it is difficult to have a comprehensive understanding of right PDH. Combining cases encountered in clinical practice and cases mentioned in previous reports, here we classified right PDH into three types according to anatomical and etiological features and then summarized surgical methods for the treatment of right PDH.

Right PDH is a part of or the entire small intestine entrapped in Waldeyer's fossa due to congenital factors. It is located laterally and inferiorly to the descending part of the duodenum. The mesentery of the ascending colon forms the anterior wall, within which lie ileocolic and right colic vessels. The posterior wall is formed by the posterior parietal peritoneum. The inner and lateral boundaries are the superior mesenteric vessels and lateral attachments of the right colon. There are additional anatomical characteristics of right PDH that can vary significantly. Moreover, some authors use certain anatomical features as diagnostic criteria for right PDH. For example, the afferent and efferent intestinal loops of right PDH in the hernia opening are reported as tight and narrow [1, 3, 15, 48]. In other reports, features such as the third part of the duodenum not crossing the midline and the cecum remaining in the lower right quadrant are used as some criteria for the diagnosis of right PDH [14, 15, 33]. However, these anatomical features correspond to only a subset of right PDHs.

According to different anatomical features, we divided right PDH into three types. The first type of right PDH (Fig. 3A) has an intact duodenal morphology; hence, the duodenum has normal horizontal and ascending parts, and the ligament of Treitz is in its normal anatomical position [1–3, 6, 12, 13, 15, 18, 19, 22, 28, 34, 36–39, 42]. The orifice of the hernia sac is located in the jejuno-cecolic isthmus, dorsally to the superior mesenteric vessels. The hernia contents mainly include the small intestine. Both afferent and efferent loops pass through the hernia orifice. From an etiological standpoint, there is no evidence of midgut malrotation in this type of right PDH. The main pathogenesis of the first type of right PDH might be an inadequate fusion of ascending mesocolon and retroperitoneum, resulting in the formation of Waldeyer’s fossa. This fits Moynihan’s theory [49] and is supported by other literature [13, 50]. Depending on the location and size of the opening of the hernia sac and the amount of hernia contents, hernia contents might return to their normal position by themselves after postural changes or treatment in some patients. Clinically, these patients have recurrent abdominal pain lasting for several years but no obvious abnormalities on CT or gastrointestinal angiography.

According to previously reported cases and the case reported in this article, the common anatomic features of the second type of right PDH are the passage of only the efferent loop through the hernia orifice and the absence of the ligament of Treitz [14, 16, 20, 21, 23–25, 27, 29, 32, 33, 35, 40, 41, 43, 44] (Fig. 3B). Absence of the ligament of Treitz means that the Treitz’s angle is located to the right of the mesenteric vessels. On imaging, the horizontal portion of the duodenum is short and not evident in some patients (Fig. 1B). In others, the horizontal portion of the duodenum runs close to the mesenteric vessel but does not pass behind the root of the mesenteric vessels. The horizontal part of the duodenum passes directly into the hernia sac. The pathogenesis of this kind of right PDH is consistent with the hypothesis proposed by Andrews in 1923 [51]. In his opinion, right PDH is the result of poor or incomplete rotation of the prearterial limb of the midgut loop [51]. The rotation of the posterior arterial limb is normal, which is consistent with the normally located cecum. In this type of right PDH, it is necessary to pay attention to the change of the orifice position of the hernia sac. Different positions require different treatment methods.

The typical anatomical features of the third type of right PDH are based on the second type of right PDH. Specifically, the Ladd’s bundle extends from the abnormal site of the cecum to the peritoneum or liver, fixing the cecum in an abnormal position, usually in the right upper quadrant (Fig. 3C) [17, 26, 30, 31]. During this process, the Ladd’s ligament crosses and compresses the duodenum or small intestine, causing intestinal obstruction. There is usually an adhesion between the mesenteric membrane of the jejunal loop and the mesenteric membrane of the ileal loop (mesenteric fusion of Pellerin). In this case, the rotation of pre- and postarterial segments is incomplete, which is a view different from Andrews’ theory [51] and supported by other literature [30].

Different anatomic features and etiologies may require different surgical methods. It is unrealistic to pursue the same surgical procedures in all types of right PDH. In the selection of surgical methods to treat PDH, a surgeon should follow the general principles of surgical treatment of abdominal hernia and then choose different surgical methods according to different anatomical characteristics. The surgical treatment of internal hernia is based on the principle of hernia reduction, repair of the defect, or enlargement of the hernia opening [49]. Specifically for right PDH, the appropriate surgical method should be selected according to its anatomical characteristics.

In the first type of right PDH, the rotation of pre- and postarterial segments is normal, and the main cause of this disease is the fusion defect of the ascending mesocolon with the posterior peritoneal wall. For type I right PDH, in Table 1, closing the orifice was chosen in five cases [18, 34, 36, 39, 42], and enlarging the orifice was chosen in five cases. The surgical plan for expanding the hernia orifice included the Ladd’s procedure(s) (the lateral approach [Fig. 4A]) in three cases [6, 12, 28] and opening the avascular area dorsal to the superior mesenteric vessels in one case [19], similar to the plan shown in Fig. 4B. Another case involved the incision of the lower peritoneal reflection of the hernia orifice below and parallel to the superior mesenteric vessels [38]. Surgical methods used were unknown in four cases. On the basis of the theory of defective fusion of retroperitoneal tissue with the ascending mesocolon, closure of the hernia sac opening should be preferred to avoid re-herniation of the small bowel, as is done in left PDH. This method will not create a new separation surface in the abdominal cavity, reducing the possibility of postoperative adhesions. Furthermore, this surgical approach is supported by the literature [13, 39]. However, in patients with severe infection and severe edema of peritoneal and posterior peritoneal tissue, suturing might not be an appropriate approach due to possible suture incompleteness, in which tissue tears where the sutures were placed might cause a secondary hernia of the small intestine and injury to the superior mesenteric vessels. Thus, dilation of the hernial sac is a reasonable option in this setting. According to Table 1, there are currently three scenarios for enlarging the orifice. Among them, the lateral approach was used in three cases [6, 12, 28] without serious complications reported in the literature. However, in the literature using this protocol, the cause of type I right PDH is attributed to intestinal malrotation. Additionally, this surgical method completely destroys the normal anatomy in the abdominal cavity and completely frees the ascending colon and cecum. Finally, there are certain disadvantages, such as a large separation surface. The protocol adopted in our case (Fig. 4B) does not have this disadvantage and can achieve the purpose of surgery while reducing trauma and maintaining the original anatomy in the abdominal cavity.

For type II right PDH, the main etiological features are the absence of the ligament of Treitz, the presence of only the efferent intestinal loops in the hernia orifice, and normal rotation of the postarterial limb of the midgut loop. We suggest that the ideal approach for type II right PDH is to fully dilate the hernia orifice. The choice of surgical regimen for type II right PDH presented in Table 1 can lead to the same conclusion. There are two ways to expand the hernia sac opening: the medial approach and the lateral approach (Fig. 4A). The case reported here included a complete opening of the avascular area dorsal to the superior mesenteric vessels (Fig. 4B). The case reported by Lee *et al*. used a similar surgical approach [43]. The lateral approach is described in several articles [20, 25, 27, 29, 32, 40, 41], and similar to the above-mentioned method, divides the lateral peritoneal folds of the right colon and then dilates the hernia orifice. Considering the obtained results, this surgical method can indeed achieve the purpose of surgery. In a special case of this type, the hernia orifice opens at the root of the superior mesenteric vessel (Position H, Fig. 3B) [16, 24, 33, 35, 44]. Some authors reduce the contents of the hernia and narrow the orifice of the hernia sac up to the point that the proximal jejunum can just pass through [24, 33, 35, 44]. This operative approach has two drawbacks. First, due to the absence of Treitz’s ligament, this type of right PDH lacks binding force to the proximal jejunum, which might re-herniate into the hernia sac; therefore, some authors use an absorbable mesh to immobilize the proximal jejunum [44]. Second, there is also a report of secondary operations due to superior mesenteric artery (SMA) compression syndrome [16]. The intestinal clusters that pass through the hernia orifice into the sac seem to lift up the SMA into the ventral position, which masks the true angle of the SMA relative to the abdominal aorta [16]. Therefore, considering that PDH is mostly performed in an emergency, it is difficult to assess the true angle of the SMA relative to the abdominal aorta before and during surgery in this particular type of right PDH. Furthermore, the obstruction is located in the efferent loop. Thus, the medial approach (Fig. 4B) might be more appropriate.

For type III PDH, the Ladd’s procedure is a standard protocol. First, the Ladd’s bands are cut off, and the ascending colon is freed from the greater omentum and the greater curvature of the stomach, allowing the ileocecal junction to be placed on the left lower quadrant. The Treitz’s angle is then loosened to avoid twist and angulation of the duodenum. Afterward, any adhesions between the mesentery of the first jejunal loop and that of the last ileal loop (mesenteric fusion of Pellerin) are removed [52, 53]. Finally, the jejunum is located in the upper right abdomen, and the ileocecal junction is located in the lower left abdomen.

**Fig. 5:**
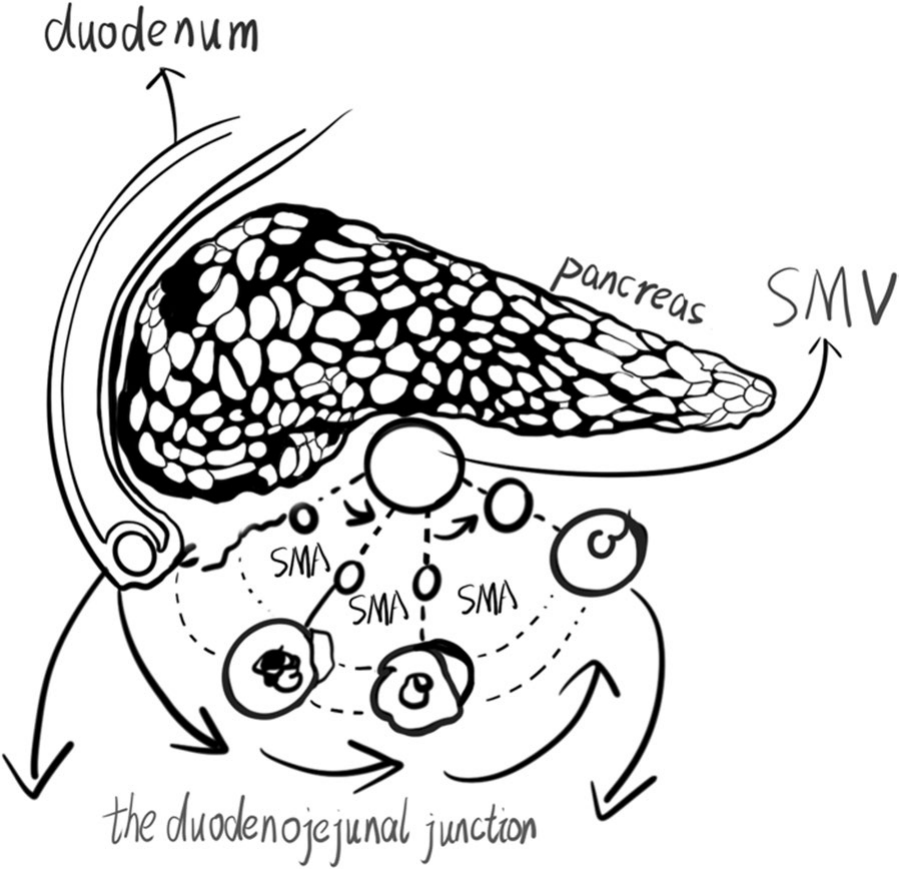
The SMA appearing to revolve around the SMV on the basis of the location of the duodenojejunal junction

The classification of right PDH can also be described from the perspective of embryonic development. The final location of the gut is related to two factors. First, the duodenum grows longitudinally during development, pushing the duodenojejunal junction dorsally to the superior mesenteric vessels and eventually to the left side of the vertebral column [54]. This process is essential for the proper alignment of the gut within the abdominal cavity. In multiple literatures [17, 20, 23, 25, 30, 35, 40] as well as the case reported in this study, the relative positions of the duodenojejunal junction, SMV, and SMA further substantiate this process. The SMA appears to revolve around the SMV on the basis of the location of the duodenojejunal junction (Fig. 5). Second, the small intestine develops rapidly within the umbilical cord and then pushes the cecum to a different location [54]. This suggests that the final position of the cecum is actually a passive result. Thus, the above-described findings provide a basis for the classification of right PDH from the perspective of embryonic development. The first type of right PDH is associated with problems with the fusion of the ascending mesocolon and the dorsal peritoneum, while the second type occurs due to failure to push the duodenojejunal junction into its normal position during early duodenal development. The third type superimposes the abnormality of the position of the cecum on the basis of the second type.

From the above-presented observations of embryonic development, we can further discuss the surgical approaches. We believe that for type I right PDH, the incomplete fusion of the ascending mesentery and the dorsal retroperitoneum needs to be resolved. For type II right PDH, the purpose of surgery is to solve the problem that the horizontal part of the duodenum cannot pass through the dorsal side of the superior mesenteric vessel to reach the left side of the vertebral column during embryonic development. To achieve this purpose, medial and lateral approaches can be adopted. At present, the lateral approach is the most used in the literature. The lateral approach is considered to be a simple Ladd’s procedure, which does not release the right colon from the hepaticocolic ligament and the greater curvature of the stomach and fails to achieve the purpose of inverting the right colon. The lateral approach only opens the hernia orifice from the outside. This view is also supported by other authors [29, 55]. For type III right PDH, the best solution is the Ladd’s procedure, which is also indisputable.

## Conclusion

This paper classified right PDH according to anatomical features and embryonic development. Additionally, the surgical methods for different types of right PDH were summarized. The authors believe that this paper will help surgeons improve their understanding of right PDH in their clinical work and choose a rational surgical approach.

## Data Availability

Not applicable.
